# Synthesis of bio-oil from waste *Trichosanthes cucumerina seeds*: a substitute for conventional fuel

**DOI:** 10.1038/s41598-020-74130-9

**Published:** 2020-10-20

**Authors:** Rajayokkiam Manimaran, Kandasamy Murugu Mohan Kumar, Nagarajan Sathiya Narayanan

**Affiliations:** School of Mechanical Engineering, SASTRA Deemed University, Tanjavur, Tamil Nadu 613 401 India

**Keywords:** Renewable energy, Mechanical engineering, Biodiesel

## Abstract

The present study explores the methodology for the synthesis of bio-oil from waste trichosanthes cucumerina seeds by the solvent extraction method. It investigates the yield percentage, concentration of free fatty acids and acid contents in the extracted bio-oil. Effects of size of the crushed seeds, moisture content, extraction time, solvent to seed ratio and extraction temperatures were examined. The non-polar hexane solvent resulted in a higher percentage of oil yield (28.4 ± 0.4%) for the crushed seed size of 0.21 mm, 6% moisture content, 270 min extraction time, 68 °C temperature and 6:1(ml/g) of solvent to seed ratio. The synthesized bio-oil was characterized using Fourier Transform Infra-Red spectrum and Gas Chromatography–Mass Spectroscopy analysis. The properties of the bio-oil and biodiesel were assessed according to the American Society for Testing and Materials and the Association of Official Analytical Chemists standards. The obtained methyl-ester by trans-esterification process results in the fuel properties closer to the conventional fuel. Thus, Trichosanthes cucumerina bio-diesel can be used as a potential substitute.

## Introduction

The increase in the percentage of energy consumption by various sectors makes the fossil sources in deficiency and makes the researchers start to shift to renewable energy sources^1^. Among the various alternative sources, straight vegetable oil has identical fuel properties, sulphur-free and bio-degradable, which makes it as the substitute for fossil fuels^2^. The bio-diesel from renewable sources shown positive impacts for better engine performances and cleaner emissions in the automotive sector^3^. In general, the vegetable oils are classified as edible and non-edible feedstocks. The edible feedstocks namely coconut, olive, palm, peanut, rice bran, soybean and sunflower are the predominant in the production of bio-diesel but leads to risk in food supplies, bio-diversity and increase the cost of the fuel. Therefore, the non-edible feedstocks like cottonseed, jatropha, jojoba, polanga, karanja, linseed, mahua, neem, rubber seed and tobacco were considered as the substitutes^4,5^.

The bio-origin materials produce a variety of biofuels through the conversion routes of pressing, extraction, chemical processes (hydrolysis and trans-esterification), biochemical processes (fermentation and anaerobic digestion), thermochemical processes (flash pyrolysis, gasification and hydrothermal liquefaction) and direct combustion^6^. The reactive extraction is considered as the most viable technology in the production of bio-diesel compared with other methods. The reactive extraction based soxhlet extractor combines the oil extraction and transesterification processes used for the bio-oil production using various seeds^7,8^. Crotalaria juncea oil, jojoba oil, zanthoxylum bungeanum oil, jatropha oil, indigofera colutea oil, soybean oil, senna occidentalis oil, cassia javanica oil and palm oil were produced with the soxhlet extractor and reported in the literature^9,10^.

In the trans-esterification process, the traces of free fatty acids in the oil extracted gets removed and produce the by-products of ester and crude glycerol with the assistance of an acid catalyst, alkali catalyst and purification process^11–13^. This trans-esterification process is considered as the most feasible and commercially used technique for the conversion of methyl esters and effective reduction of viscosity for the bio-oils^14^. The physicochemical properties such as cetane number, heating value, kinematic viscosity, density, flash and fire points of the bio-diesel obtained using trans-esterification processes were assessed with ASTM standard methods^15^.

The bio-oil extraction from jatropha curcas seeds was studied with the functional parameters of solvent namely extraction temperature, type of solvent used (n-hexane and petroleum ether), solvent-to-seed ratio, extraction time and particle size of the seeds. The n-hexane solvent was seen with the maximum efficiency and 1.3% higher when compared to petroleum ether for the optimum condition^16^. The separation of bio-oil from urban waste putranjiva roxbughii seeds were carried out with the five different solvents (Diethyl ether, Hexane, Isopropanol, Toluene and Chloroform). The non-polar solvent hexane seen with better results compared to other considered solvents and also reported that the higher boiling point of toluene requires more heat to form vapour during the solvent recovery distillation process compared to hexane^17^. The non-polar solvents (toluene, chloroform and n-hexane) produced maximum bio-oil yield compared to polar solvents (methanol and 2-propanol) in the extraction of bio-oil from date palm seeds^18^.

This research is focussed on the synthesis of bio-oil extraction from the waste trichosanthes cucumerina seeds and its fundamental properties were investigated. Further, the trans-esterification process was carried out to remove the free fatty acids in the bio-oil and converted to bio-diesel. The trichosanthes cucumerina biodiesel was obtained from trans-esterification processes with effective fuel properties. The presence of functional groups and the free fatty acid content were characterized using GC–MS and FTIR analysis. The properties of trichosanthes cucumerina bio-oil and bio-diesel were measured using the AOAC and ASTM standard methods and compared with the conventional fuel.

## Materials and methods

### Trichosanthes cucumerina seed and solvents

**Figure 1:**
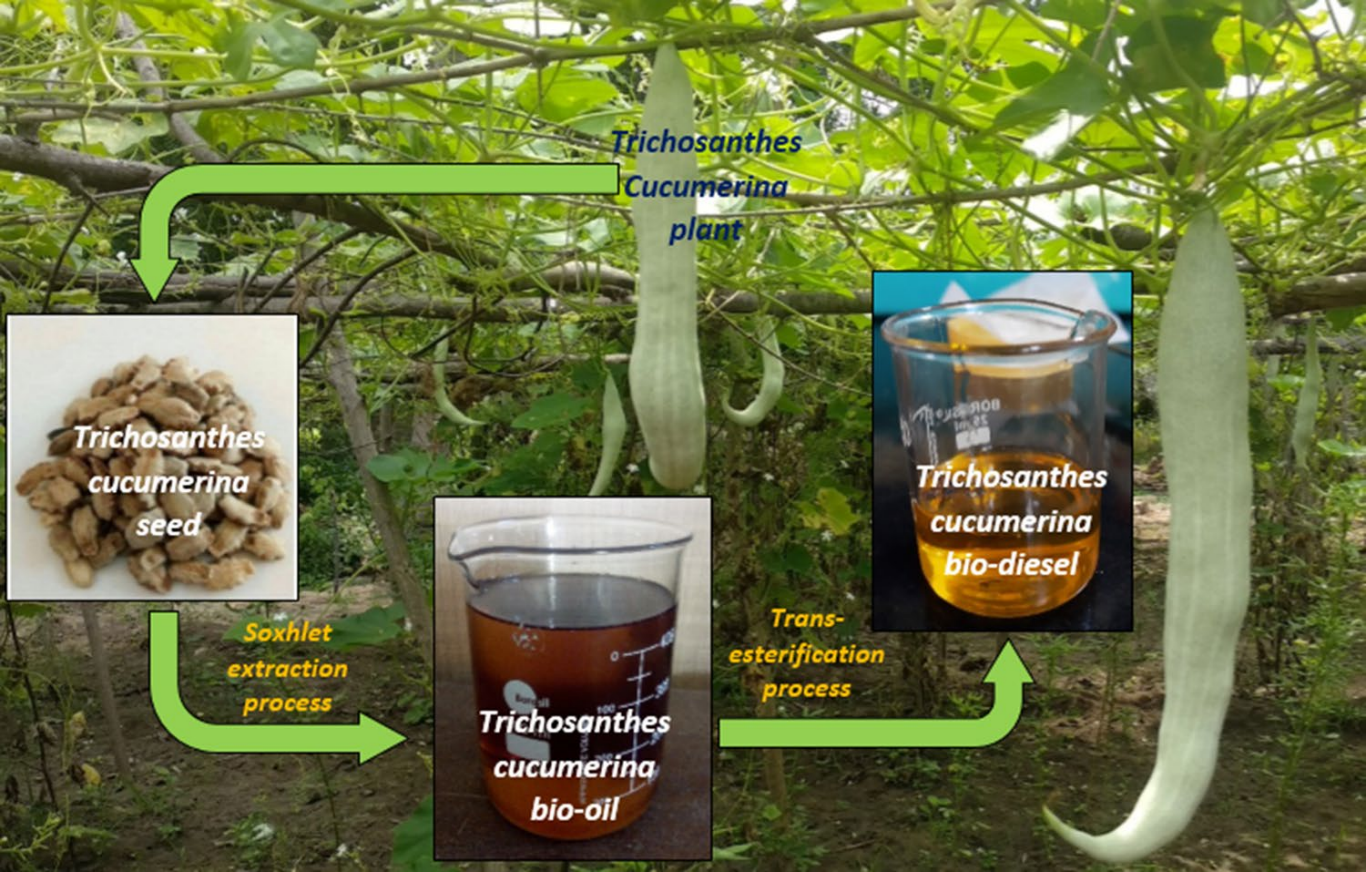
Trichosanthes cucumerina fruit, waste seed, TCO and TCB. It presents the image of Trichosanthes cucumerina fruit and its collection of waste seed. Also, the extraction of bio-oil and production of bio-diesel.

The trichosanthes cucumerina is an oil-rich seed plant that comes under the family of curcubitaceae. It originated in Asian countries and referred as snake gourd, viper gourd and snake tomato. Figure 1 shows the trichosanthes cucumerina which are 40–120 cm long, pale-green in colour, 0.5–1 kg weight (single fruit) and contains 40–70 seeds. Seeds are received from the local market and segregated for the bio-oil production. Five solvents namely polar protic (methanol, ethanol and isopropanol), dipolar aprotic (acetone) and non-polar (hexane) purchased from SRL Chemicals Pvt Ltd, Mumbai, India. The physical properties of the different solvents are listed in Table 1.

**Table 1:** Properties of different solvents used^18–21^.

Solvent parameters	Solvents used				
	Acetone	Methanol	Hexane	Ethanol	Isopropanol
Chemical formula	C_3_H_6_O	CH_3_OH	C_6_H_14_	C_2_H_5_OH	C_3_H_7_OH
Boiling point (°C)	56.1	64.5	68.7	78.3	82.3
Density (kg/m^3^)	784	791.4	655	789.3	786
Refractive index at 20 °C	1.36	1.33	1.375	1.36	1.376
Dielectric Constant at 20 °C	20.7	32.7	1.88	24.5	18.6
Latent heat of vaporization (kJ/kg)	511	1100	335	836	666
Viscosity at 20 °C (mPa-s)	0.33	0.593	0.32	1.19	2.37
Surface tension at 20 °C (mN/m)	25.20	22.70	18.43	22.10	23

### Bio-oil extraction process

**Figure 2:**
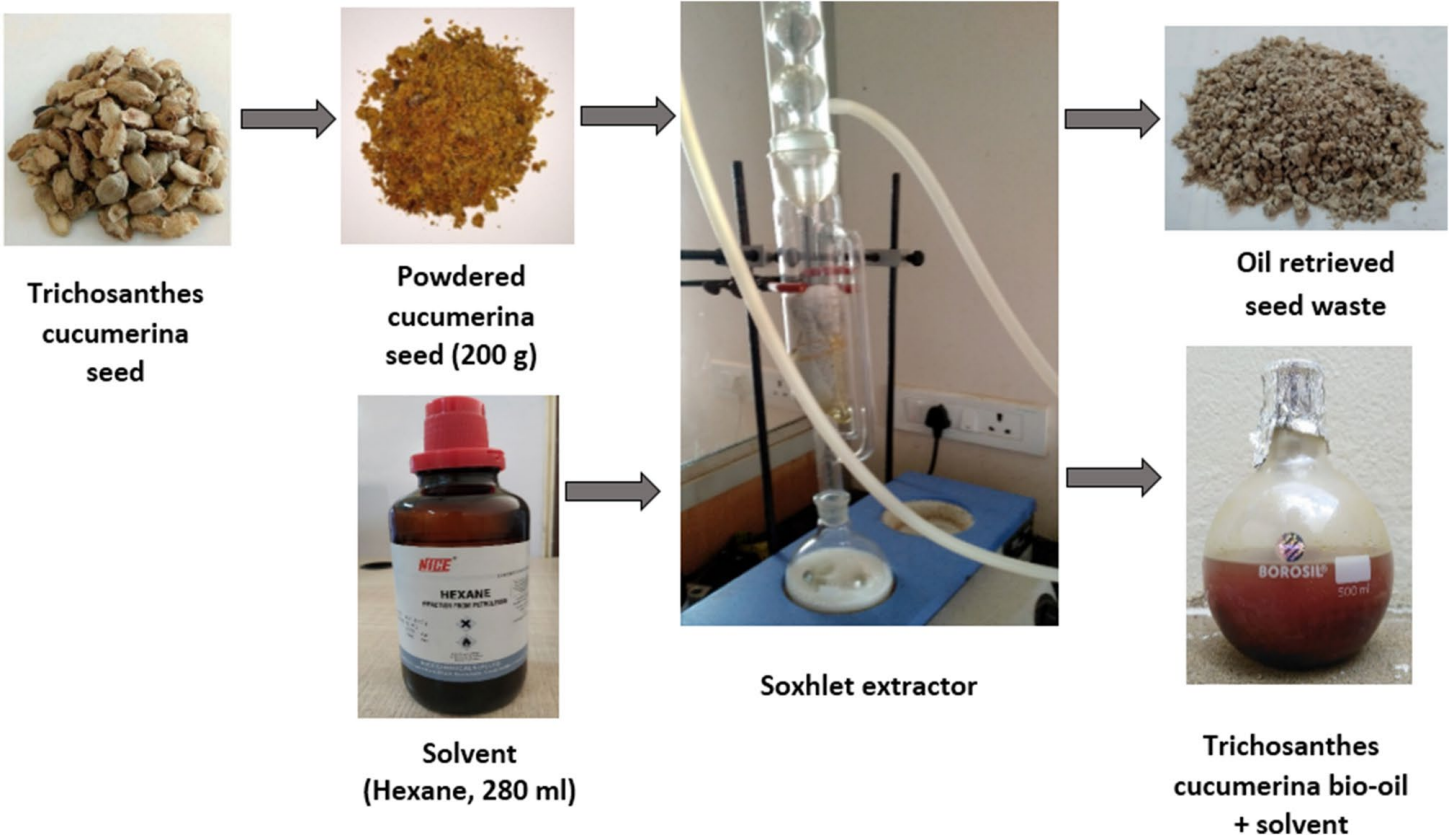
Trichosanthes cucumerina bio-oil extraction flow process. The bio-oil extraction from powdered cucumerina seed with hexane using a Soxhlet apparatus produces the oil retrieved seed waste and bio-oil associated with hexane.

**Figure 3:**
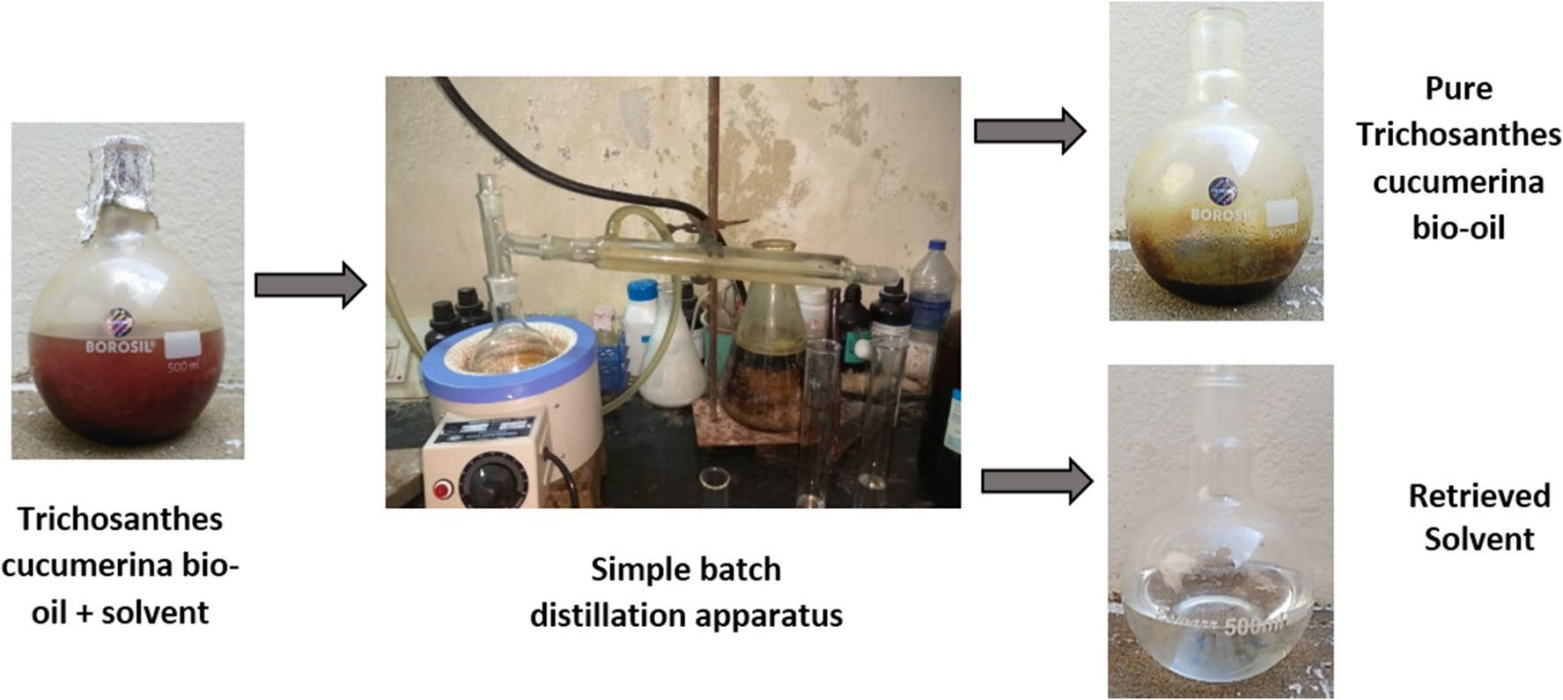
Batch distillation flow process. The mixture of bio-oil with solvent collected from Soxhlet extractor was separated through a separation process. It resulted in around 60% of the solvent, and 28.4% of bio-oil yield.

The segregated seeds were washed with distilled water (in-house) for the removal of impurities. The seeds outer layer were removed and dried in an oven at 65 °C temperature. The dried seeds were crushed in the oilseed crushing machine (SASTRA Deemed University, Tanjore, Tamil Nadu, India) and obtained with 7% of the bio-oil yield from the taken 200 g of trichosanthes cucumerina seeds. From the literature, the soxhlet extractor was selected to extract the bio-oil yield^8,9^. The round bottom flask of 500 ml capacity, filled with 280 ml of hexane and kept in the heating mantle. The powdered cucumerina seeds of 200 g were packed with a satin cloth which was held inside the thimble. A reflux condenser was attached at the top with inlet and outlet ports for cooling water circulation using aquarium motor. The heated solvent vapors were passed over the bio-mass through distillation path and cooled using a condenser. The cold vapor drips back into the chamber, which was emptied by siphoning action. It results in an extracted trichosanthes cucumerina bio-oil with solvent and the oil retrieved seed waste is depicted in Fig. 2. In batch distillation process, the obtained bio-oil and solvent mixtures were heated (25 to 85 °C) to evaporate the solvents to separate the bio-oil from the solvent as shown in Fig. 3. The experimental work was performed for three times with each solvent and the average was considered.

### Trans-esterification process

**Figure 4:**
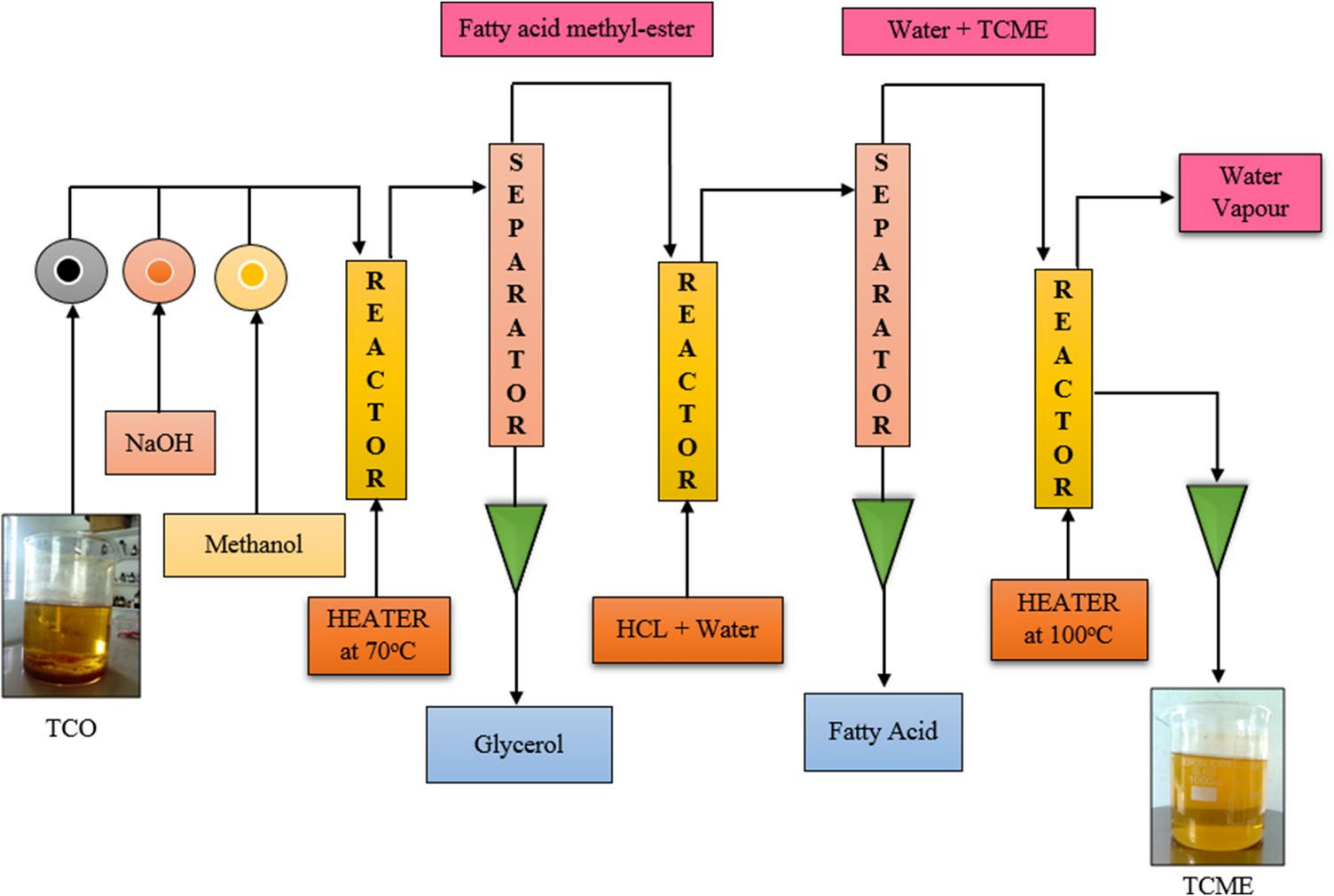
The sequence of trans-esterification process. It is the process to which triglycerides of Trichosanthes cucumerina bio-oil converted into their mono-esters by reacting them with alcohols (methanol or ethanol) in the presence of a catalyst (NaOH or KOH).

The bio-oil leached from trichosanthes cucumerina seeds found with FFA content via GC–MS test which needs the trans-esterification process. In the process, the triglycerides of trichosanthes cucumerina bio-oil converted into its mono-esters by reacting with alcohols in the presence of NaOH or KOH^18,22^. The bio-oil prepared from the seeds of trichosanthes cucumerina fruit was subjected to the transesterification process using acid-catalyzed esterification, alkali catalyzed esterification and purification stages. Initially, the bio-oil was mixed with methyl alcohol in 16:1 molar ratio then added with 1% of H_2_SO_4_. The mixture was heated at 60 °C for 45 min which reduced the acid value of bio-oil to less than 4 mg of KOH/g^23^. Next, the bio-oil (750 ml) and methyl alcohol (400 ml) were mixed with alkali catalyst (NaOH) and subjected to stirring at a constant speed of 1500 rpm for 30 min duration. The end of the process observed with fatty acid methyl ester, glycerol and the traces of NaOH. Further processing with the addition of HCL and H_2_O and followed by the purification process, the traces were removed and obtained with 93.4 ± 0.2% Trichosanthes Cucumerina Biodiesel (TCB). The trans-esterification process, as shown in Fig. 4, was carried out at SASTRA Deemed University, Tanjore, Tamil Nadu, India.

### Bio-oil yield

The percentage of bio-oil yield was calculated with the formula (1) for all the five different solvents considered in the research (methanol, ethanol, isopropanol, acetone and hexane).

1 $$\% \;of\; bio - oil \;yield^{.} = \frac{{Trichosanthes\; cucumerina \;bio - oil \; \left( {{\text{gm}}} \right)}}{{Trichosanthes\; cucumerina \;seed \;\left( {{\text{gm}}} \right)}} \times 100$$

**Figure 5:**
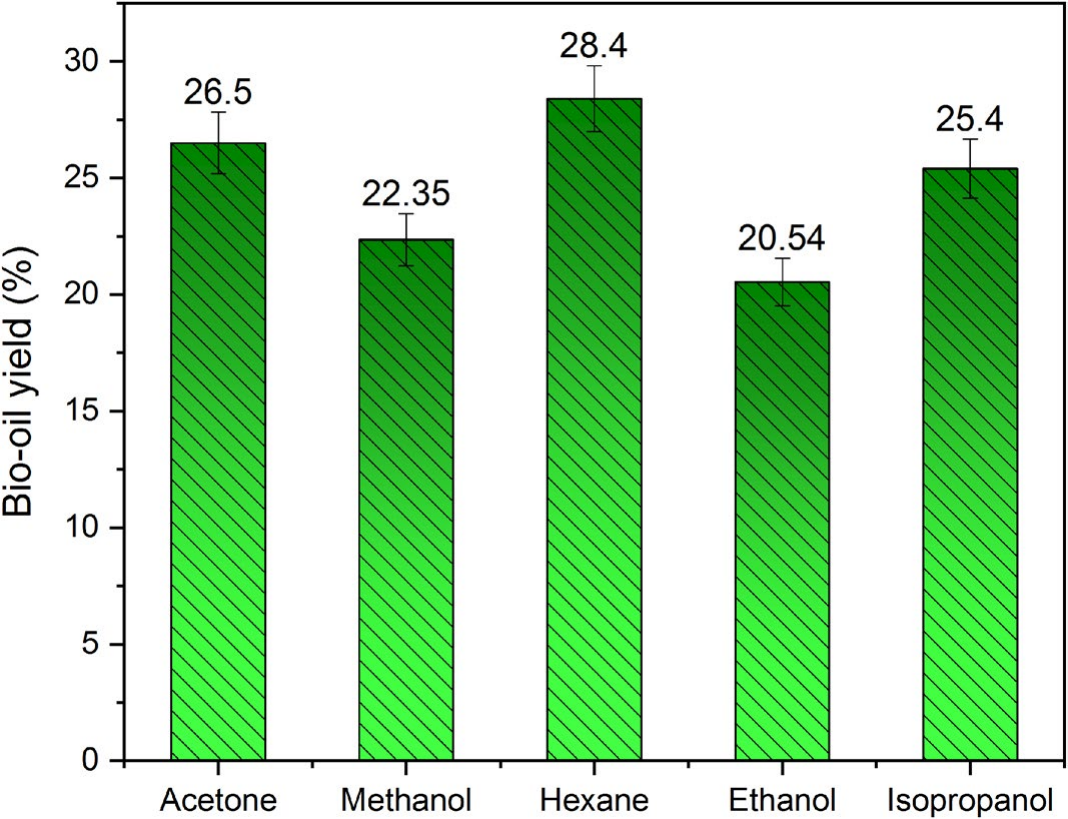
The maximum percentage of bio-oil yield with different solvents. The bar charts represent the comparison of the maximum percentage of bio-oil yield with different solvents used.

The corresponding percentage differences between the different solvents is shown in Fig. 5. It was evident that hexane resulted in a high percentage of yield was about 28.4 ± 0.4%. It may be attributed due to its low latent heat of vaporization, the minimum value of dielectric constant and surface tension, lower density and viscosity as compared with other solvents are listed in Table 1. The least percentage of 20.54 ± 0.2% was found with ethanol due to their higher polarity and solubility in water which declined the bio-oil yield*.* The results obtained in this research were in line with the results of the other researchers^8,17,20,24–27^, are reported in Table 2.

**Table 2:** Comparison of Trichosanthes cucumerina bio-oil (TCO) yield using different solvents.

Bio-oils	Bio-oil yield (%)								
	Acetone	Methanol	Hexane	Ethanol	Isopropanol	Butanol	Petroleum ether	Chloroform	Toulene
TCO [This study]	26.50 ± 0.2	22.35 ± 0.15	28.40 ± 0.4	20.54 ± 0.2	25.40 ± 0.3	–	–	–	–
ZSO^8^	–	20.2	26.3	23.4	–	–	25.3	–	–
PRSO^17^	–	–	43.07	–	40.61	–	36.9	25.6	30.5
CGAO^20^	–	8.6	11.76	–	9.8	–	–	7.6	9
ULO^24^	6.55 ± 0.04	7.51 ± 0.04	8.53 ± 0.04	7 ± 0.05	7.89 ± 0.06	–	–	–	–
SSO^25^	19.28	20.3	22.65	–	23.46	–	–	18.72	15.23
CFSO^26^	12.5	18	27.03	–	22	–	–	15.2	–
CJSO^27^	20.5	18.5	24.4	–	22.3	–	–	16.5	20.6

*ZSO* Zanthoxylum bungeanum seed oil, *SSO* Senna occidentalis seed oil, *ULO* Ulva lactuca oil, *CFSO* Cassia fistula seed oil, *CJSO* Cassia javanica seed oil, *PRSO* Putranjiva roxburghii seed oil, *CGAO* Cladophora glomerata algal oil.

## Results and Discussion

### Effect of moisture content

**Figure 6:**
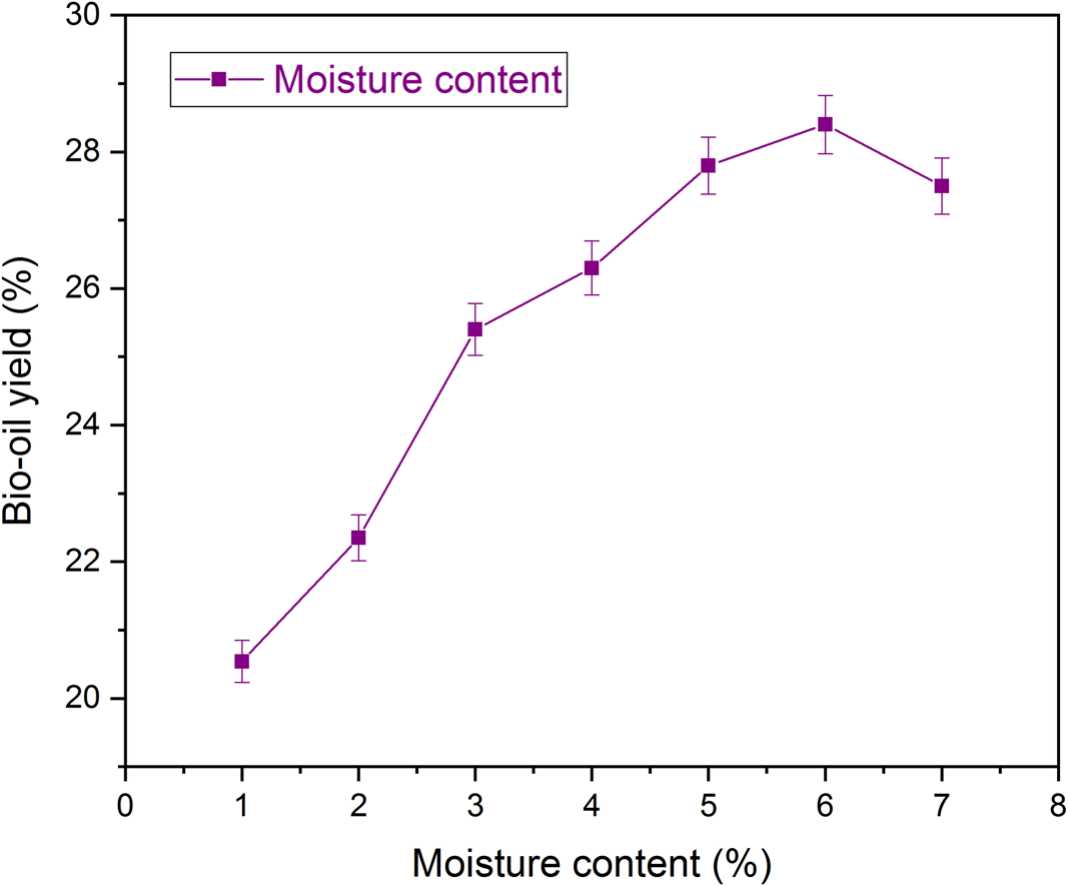
Effect of moisture content on bio-oil yield using hexane. The moisture content (%) of the seed increases with an increase in bio-oil yield with the optimum conditions of 0.21 mm seed size, 270 min extraction time, 68 °C temperature and 6:1(ml/g) of solvent to seed ratio.

The weight of the seeds with moisture and without moisture was measured using infrared moisture analyzer. For the first set of experiments, trichosanthes cucumerina seeds with the moisture content of 1 ± 0.02% were considered to check the yield of bio-oil with hexane as the solvent and resulted in 20.5 ± 0.3%. The increase in bio-oil yield was observed for the moisture content of seed between 1 ± 0.02% to 6 ± 0.02%, as shown in Fig. 6. Further increase in moisture content reduced the yield since the penetration of hexane into the trichosanthes cucumerina seed, and the higher moisture content functioned as the barrier for bio-oil extraction^28–30^. The higher percentage of yield (28.01 ± 0.3%) was observed at 6% of moisture content, by keeping other parameters as constant (size of seed as 0.21 mm, extraction time of 270 min, at 68 °C and the solvent-to-seed ratio of 6:1 ml/g hexane). The further increase in the percentage of moisture content shows in the reduction of bio-oil yield. Farsie and Singh^31^ reported that the maximum percentage of bio-oil yield was obtained from sunflower seeds expressed at 6% of moisture content. Muhammad Muhammad Fadhlullah et al.^32^ determined the effect of moisture content in the bio-oil generation using Calophyllum inophyllum L. seeds. It was observed that the yield was increased from 28.87% to 33.39% for the moisture content of the seeds of 0% and 1.2% respectively. In contrast, the increase in moisture content to 20% the yield gets reduced to 15.56%. Orhevba et al.^33^ experimented with 6.3, 8.1, 13.2 and 16.6% of moisture content of neem seed kernel and observed with 22.3, 24.86, 21.21 and 15.62% of bio-yield respectively. The maximum percentage of bio-oil yield of 24.86% was observed at the optimum moisture of 8.1% while the least yield of 15.62% was recorded at the highest moisture content of 16.6%. Suganya and Renganathan^24^ carried out bio-oil extraction from marine macroalgae Ulva Lactuca with 12 different solvents. The authors observed the maximum bio-oil yield at 5% of moisture content with hexane as solvent after with 5% the yield gets declined.

### Effect of seed size

**Figure 7:**
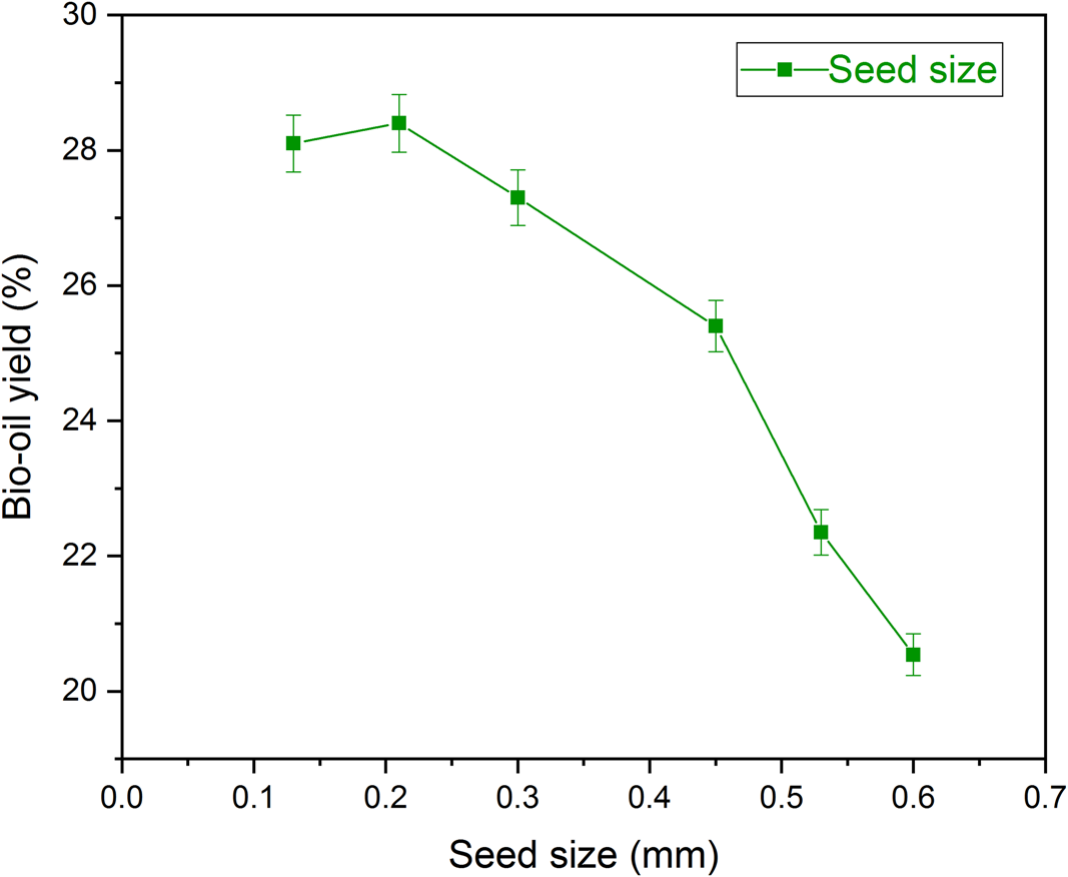
Effect of seed size on bio-oil yield using hexane. The crushed seed size (mm) decreases with an increase in bio-oil yield (optimum conditions: 6% moisture content, 270 min extraction time, 68 °C temperature and 6:1(ml/g) of solvent to seed ratio.

The extraction of bio-oil was carried out with different sizes of the seed in the range 0.6 ± 0.02 mm to 0.15 ± 0.02 mm were present in Fig. 7. Initially, the seed of size 0.6 ± 0.02 mm was considered for the experimentation with hexane as a solvent which resulted in 20.54 ± 0.3% of bio-oil yield. Further experiments have seen with an increasing trend of yield till 0.21 ± 0.02 mm size of the seed (bio-oil yield as 28.30 ± 0.4%) after which for the seed size of 0.15 ± 0.02 mm, the bio-oil yield get declined. Thereby the seed size seems to be the next critical parameter for the bio-oil yield extraction. The increase in contact between the solvent and seed, and mass transfer of seed (solid-state) to solvent (liquid state) shows that the decrease in the size of the seeds increased the bio-oil yield^34^. Qian et al.^35^ reported that yield from cottonseed gets increased with the reduction of particle size; in contrast, the further reduction has not shown the improvement in the yield extraction. The decrease in seed size would not always increase the yield due to the range limit, which helps in optimizing the yield^32^. The results obtained in this work were in line with the reported works of literature, the Ulva Lactuca reported with a high yield of 10.9% with the seed size of 0.15 mm^29^ and Adenanthera pavonina seen with a high yield of 26.2% for the particle size of 0.25 mm^25^.

### Effect of extraction time

**Figure 8:**
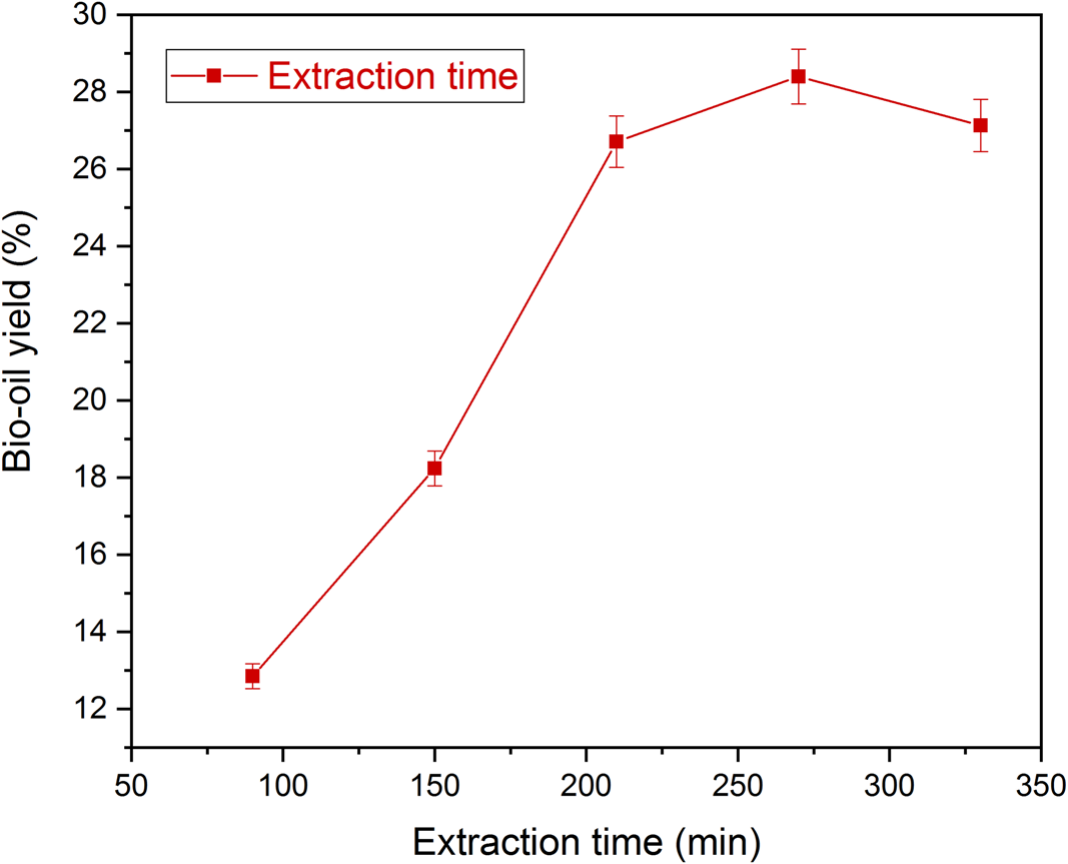
Effect of extraction time on bio-oil yield using hexane as a solvent. The bio-oil extraction from Trichosanthes cucumerina seed using hexane with the impact of time, as shown in the graph. The hexane produces maximum bio-oil yield (28.15 ± 0.3%) as compared with other solvents at an optimum extraction time of 270 min.

The effect of extraction time at different time intervals of 90,150,210,270 and 330 min for the bio-yield was examined. Figure 8 illustrates that the increase in extraction time increased the bio-oil yield from 90 to 270 min, a further increase in extraction time decreased the yield (say at 330 min). The bio-oil extraction time increased gradually, which also increases the yield percentage up to a certain extent, after that the yield reaches a plateau at longer time duration^36^. Hence the extraction time 270 min was remarked to be the optimum time for further examinations. From the literature, it was observed that the Senna occidentalis seeds produced the maximum oil yield 23.46% with the optimum extraction time of 210 min^37^. Theresa et al.^38^ investigated the extraction time between 30 to 300 min using Indigofera colutea seeds with hexane as the solvent and seen with maximum yield (38.45%) obtained at 210 min, further extension of time decreased the bio-oil yield.

### Effect of solvent-to-seed ratio

The effect of solvent-to-seed ratio was also explored by varying the ratio between 2:1 to 7:1, as given in Table 3. The increase in the bio-oil yield percentage was observed until 6:1, a further increase in ratio reduced the yield percentage. The increase in solvent volume significantly improved the yield until the value of the equilibrium reached, a further increase in solvent volume not seen with any improvement^29^. Suganya and Renganathan^24^ experimented on marine macroalgae Ulva Lactuca with the solvent-to-seed ratio varied from 3:1 to 6:1, which increased the bio-oil yield from 9% to 10.88%. The yield gets reduced for the further increase of solvent volume.

**Table 3:** Effects of solvent-to-seed (ml/g) ration and temperature (°C) on bio-oil yield extraction.

Solvent-to-seed ratio (ml/g)	Bio-oil yield (%)	Extraction temperature (°C)	Bio-oil yield (%)
2:1	12.85 ± 0.1	28	18.34 ± 0.15
3:1	20.35 ± 0.2	38	21.05 ± 0.2
4:1	22.35 ± 0.2	48	23.74 ± 0.2
5:1	26.5 ± 0.3	58	26.8 ± 0.3
6:1	28.4 ± 0.4	68	27.81 ± 0.4
7:1	26.2 ± 0.3	78	25.3 ± 0.3

### Effect of extraction temperature

The variation of extraction temperature on the bio-oil yield obtained from Trichosanthes cucumerina seed with all solvents was conducted over the range of 28 to 78 °C. The increase in bio-oil yield (hexane as solvent) from 18.34 ± 0.15% to 25.3 ± 0.3 was obtained as given in Table 3. The rise in extraction temperature increases the bio-oil yield, due to the mass transfer rate and solubilization of hexane. The lower viscosity (0.32 mPa s) and surface tension (18.43 mN/m) of hexane which improves the diffusivity and solubilization inside the solid matrix at higher temperature resulted in maximum extraction rate. The solvent dissolution capacity also would increase the bio-oil yield. On the further rise in temperature beyond 68 °C (boiling point of hexane), the bio-oil yield content decreased^39^. This rise in temperature increased the solvent boil off and reduced the active contact area between solid and liquid phases^40^. The hexane provided the maximum amount of oil extraction (27.81 ± 0.4%) with the optimum temperature of 68 °C. It was detected that the solubility of the solvent increased with an increase in the diffusion rate^41^. Sayyar et al.^16^ considered the role of extraction temperature, the higher percentage of bio-oil yield of 47.3% from Jatropha curcas using hexane as the solvent at 68 °C. Milan D. Kostic et al.^42^ optimized the extraction of bio-oil from hemp seeds using n-hexane solvent. The results show that the extraction temperature of n-hexane (at 70 °C) increased the hemp seed oil yield because of improved oil solubility in the solvent. Karthikeyan Murugesan and Renganathan Sahadevan^27^ optimized the non-edible bio-oil extraction from Cassia javanica seeds using different solvents. The hexane was the best extractor to extract the maximum percentage of bio-oil yield was about 24.4% with the optimum conditions (extraction temperature at 68 °C and extraction time at 3.5 h).

### GC–MS Analysis

**Figure 9:**
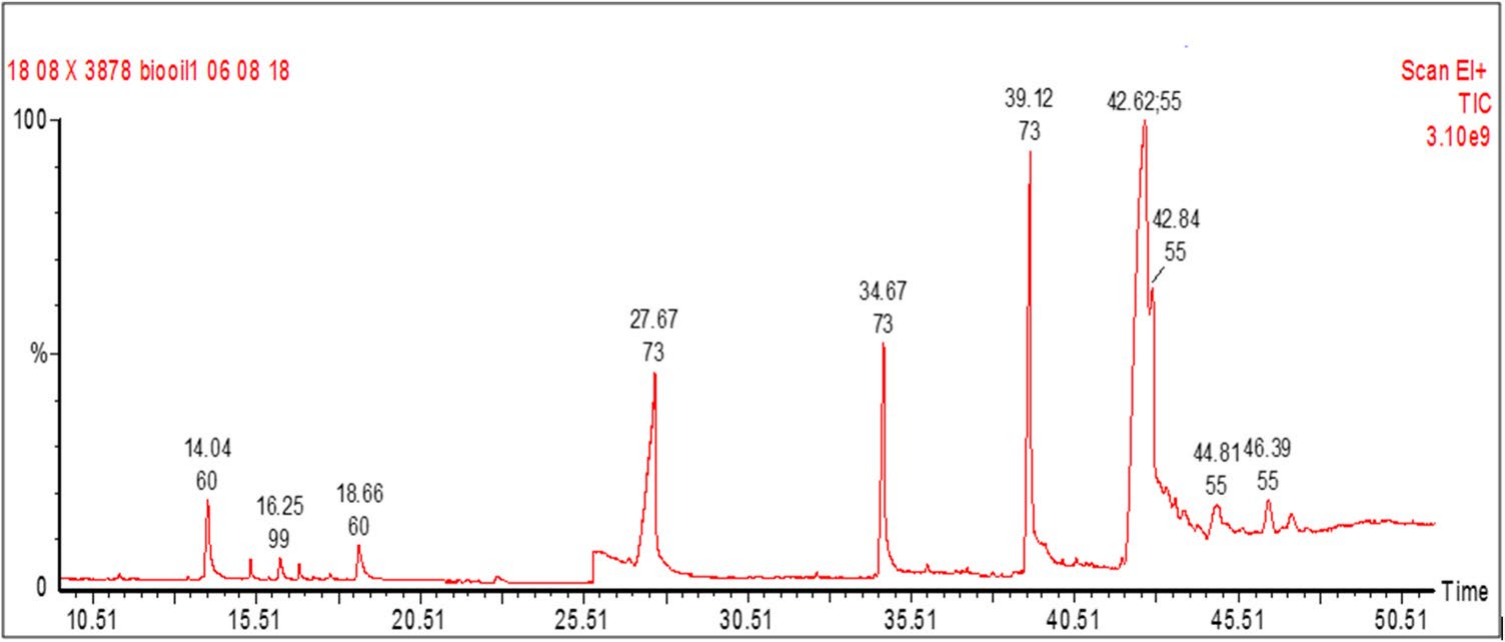
GC–MS Chromatogram of Trichosanthes cucumerina bio-oil. The trichosanthes cucumerina bio-oil organic compounds were measured by the test method of gas chromatography. It found twenty-two free fatty acids were present in the trichosanthes cucumerina bio-oil. It results in the percentage of unsaturated fatty acids as high as saturated fatty acids.

The GC–MS test analysis (refer Table 4) was performed to measure the various factors of bio-oil, presence of FFA composition and different types of hydrocarbons^43,44^. The trichosanthes cucumerina bio-oil organic compounds were measured by the test method of gas chromatography (Fig. 9), model PerkinElmer Clarus 500 coupled with a mass spectrometer. The Capillary Column Elite-5MS was used for the separation of components in bio-oil fraction for the length of 30 m. The flow rate of carrier gas fixed at 1 mL/min and the GC was operated at 58.3 min for the helium flow rate of 1 mL/min. The temperature ranges from 150 to 280 °C at 10 °C /min was maintained in the column. A sample volume of 1.0µL trichosanthes cucumerina bio-oil in chloroform was injected through a split mode, with 1:10 split ratio. The MS condition for the mass range was set to 40 to 450 amu with the complete mode of electron ionization with the electron energy of 70 eV. The components in the sample have been identified using Turbomass ver 5.2.0 software and NIST 2005 mass spectral library. From the GC–MS analysis, 22 free fatty acids were found as listed in Table 4. The oleic acid (C_18_H_34_O_2_) contributed 43.27%, dodecanoic acid (C_12_H_24_O_2_) with 13.14%, n-Hexadecanoic acid (C_16_H_32_O_2_) with 13.21%, Octadecanoic acid (C_18_H_36_O_2_) with 12.3% of the total composition percentage. Among them, 61.14% of unsaturated and 34.09% of saturated fatty acids were present in the trichosanthes cucumerina bio-oil.

**Table 4:** Fatty acid composition of Trichosanthes cucumerina bio-oil through GC–MS analysis.

Fatty acids	Molecular formula	Molecular weight	Retention time	%Peak area
Hexanoic acid	C_6_H_12_O_2_	116	9.31	0.2581
Nonanoic acid	C_9_H_18_O_2_	158	11.71	0.0418
Undecanoic acid, ethyl ester	C_13_H_26_O_2_	214	13.85	0.0806
Octanoic Acid	C_8_H_16_O_2_	144	14.04	2.7896
2-Decenal, (E)-	C_10_H_18_O	154	15.34	0.3680
2-Dodecanone	C_12_H_24_O	184	15.91	0.0403
2H-Pyran-2-one, 6-ethyltetrahydro-	C_7_H_12_O_2_	128	16.25	0.6635
2,4-Decadienal	C_10_H_16_O	152	16.82	0.3009
2H-Pyran-2-one, tetrahydro-6-propyl-	C_8_H_14_O_2_	142	22.86	0.2332
Dodecanoic acid	C_12_H_24_O_2_	200	27.67	13.1398
2H-Pyran-2-one, 6-hexyltetrahydro-	C_11_H_20_O_2_	184	32.62	0.1087
Decanoic acid, ethyl ester	C_12_H_24_O_2_	200	34.41	0.0549
Tetradecanoic acid	C_14_H_28_O_2_	228	34.61	7.5717
2-Nonadecanone	C_19_H_38_O	282	37.08	0.0885
Hexadecanoic acid, ethyl ester	C_18_H_36_O_2_	284	38.85	0.0460
n-Hexadecanoic acid	C_16_H_32_O_2_	256	39.12	13.2114
Oleic Acid	C_18_H_34_O_2_	282	42.62	43.2662
Octadecanoic acid	C_18_H_36_O_2_	284	42.84	12.3392
Hexadecanoic acid, 2-hydroxy-1-(hydroxymethyl)ethyl ester	C_19_H_38_O_4_	330	43.56	0.2971
Z,E-3,13-Octadecadien-1-ol	C_18_H_34_O	266	43.81	0.4736
9,12,15-Octadecatrienoic acid, (Z,Z,Z)-	C_18_H_30_O_2_	278	44.81	3.1031
9-Octadecenoic acid (Z)-, 2-hydroxy-1-(hydroxymethyl)ethyl ester	C_21_H_40_O_4_	356	46.39	1.5240

### FTIR analysis

**Figure 10:**
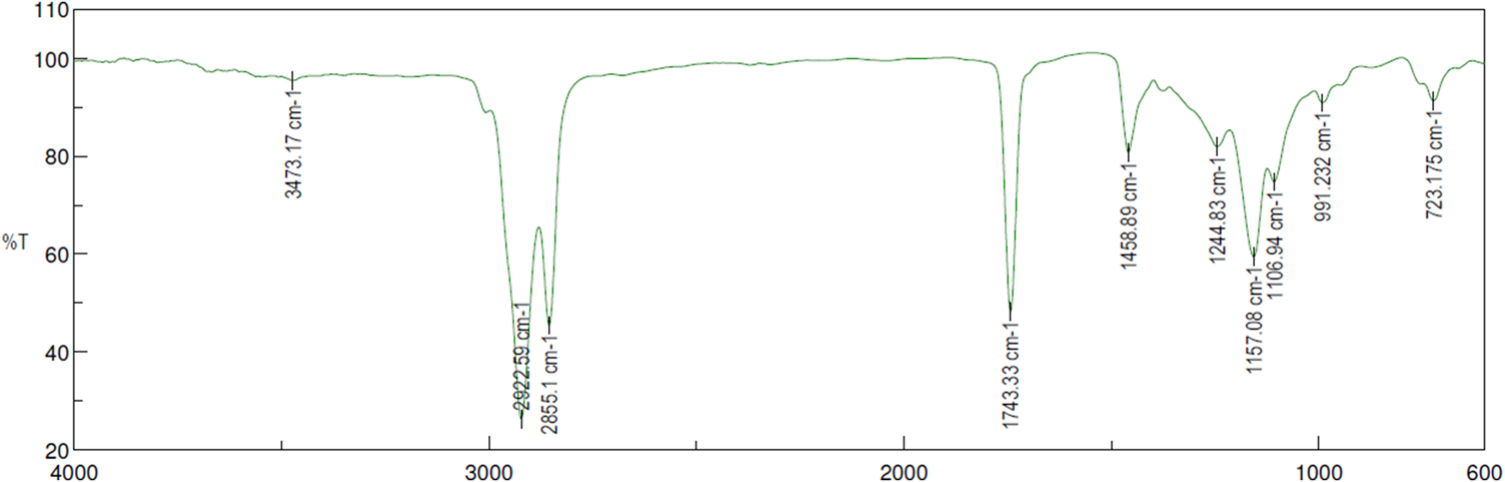
Spectra of Trichosanthes cucumerina seed oil according to FTIR analysis. The FTIR spectra were recorded between 500 cm^−1^ and 4000 cm^−1^ in the transmission mode for the trichosanthes cucumerina seed oil. The C–H stretching at 2922.59 cm^−1^and 2855.1 cm^−1^ shows the presence of alkanes. The C=C stretch proves the presence of the aromatic compounds. The presence of oxygenated functional groups (O–H, C-O and C = O) indicates the high percentage of oxygen content in the bio-oil.

The presence of polymer, organic and inorganic materials were identified through the Fourier Transform Infrared (FTIR) Spectroscopy analyzer. An infrared light source scans the test samples and analyses the chemical properties^45^. This analytical method was used to analyze the chemical bonds and its nature, based on its stretching or bending on exposure to infrared radiation. In this study, the Deuterated Tri Glycine Sulphate (DTGS) detector was used, which works on the variation in the temperature and IR radiation intensity. The FTIR spectra were recorded between 500 and 4000 cm^−1^ in the transmission mode for the trichosanthes cucumerina bio-oil, as shown in Fig. 10. The various functional groups which were identified are tabulated in Table 5. The C–H stretching at 2922.59 cm^−1^, 2855.1 cm^−1^ shows the presence of alkanes and the C=C stretch ensure the presence of the aromatic compounds^17,46^. The presence of oxygenated functional groups (O–H, C–O and C=O) indicated the high percentage of oxygen content in the bio-oil and inferred the acidic nature. The presence of hydrocarbon groups indicates the potential usage of bio-oil as the alternate source of energy^47^.

**Table 5:** Functional groups of *Trichosanthes cucumerina* bio-oil according to FTIR analysis.

Wave Number (cm^−1^)		Functional groups	Compound class	References
Theoretical Range	Experimental value			
3200–3600	3473.17	O–H Stretching	Phenols, Alcohols	^48^
2810–3000	2922.59	C–H Stretching	Aliphatics–Alkanes	
2810–3000	2855.1	C–H Stretching	Aliphatics–Alkanes	
1715–1755	1743.33	C=O Stretching	Aldehydes and Ketones	^49^
1400–1550	1458.89	C=C Stretching	Aromatic	^50^
1210–1260	1244.83	C–O Bending	Ester	^51^
1110–1210	1157.08	C–OH Stretching	Tertiary Alcohol	^47^
1080–1160	1106.94	C–O Stretching	Aliphatic ether, Secondary alcohol	^52^
950–1010	991.232	=C–H Bending	Alkene	^47^
670–810	723.175	C–H Bending	Aromatic	^49^

### Fuel properties

The fuel properties of Trichosanthes cucumerina bio-oil (TCO), Trichosanthes cucumerina biodiesel (TCB) and Diesel fuel (DF) are tested as per the AOAC and ASTM standards and carried out at Delta Inspection & Research Laboratory, Chennai, Tamil Nadu, India. The properties like kinematic viscosity (ν), fuel density (ρ), heating value, iodine number and saponification value (SV), cetane number, flash and fire points were checked and listed in Table 6.

**Table 6:** Comparison of DF, TCO and TCB fuel properties.

Fuel properties	Diesel fuel	TCO	TCB	Standard methods
Chemical formula	C_10_H_22_	C_16_H_30_O_2_	C_12_H_26_O_2_	Calculation
Molecular weight (g/mol)	142.282	252.487	203.61	Calculation
Kinematic viscosity (cSt)	2.71	42.85	4.26	ASTM D 445
Density (kg/m^3^)	836	912	856	ASTM D 4052
Heating value (MJ/kg)	42.5	32.45	38.50	ASTM D 3286
Cetane number (CN)	51	35	44	ASTM D 613
Flash Point (°C)	67	*236*	158	ASTM D 93
Fire Point (°C)	86	262	169	ASTM D 93
Acid value (mg KOH/g)	0.1	2.16	1.74	ASTM D 664
Sulphur (mg/kg)	29	–	–	ASTM D 2622
Iodine value (gI_2_/100 g)	38.3	82.4	67.2	AOAC CD1-25
Saponification value (mg KOH/g)	–	121.37	74.3	AOAC CD3-25

#### Fuel density (ρ) and kinematic viscosity (ν)

The fuel density was measured at the reference temperature of 15 °C using hydrometer and found to be 8.33% (TCO) and 6.14% (TCB) higher than diesel. The higher number of unsaturated fatty acid contents in the bio-oil increases the molecular weight. It results in higher fuel density which leads to increased compression ratio, specific fuel consumption and the rate of oxidation. The TCB density was very closer to the value of diesel fuel caused more thermal stability of the biodiesel^53,54^. The Redwood viscometer was used to measure the viscosity. The TCO obtained 42.75 cSt of kinematic viscosity which was 14.82 times higher than that of diesel which attributed to an increase in the free fatty acid chain, the breakdown of intermolecular forces and adhesion between biofuel molecules^7^. The TCB resulted in decreased kinematic viscosity and 9.05 times lesser than TCO, which improves the fuel characteristics of atomization and vaporization and provides better engine performance^55^.

#### Other properties

The other properties like fuel heating value, iodine number, saponification value, cetane number can also be referred in Table 6. The fuel heating values were numerically calculated using Dulong’s formula^56^. The higher proportion of oxygen content in the bio-oil resulted in lower heat energy (32.45 MJ/kg) as compared to diesel. The heating value of TCB moderately increased with the trans-esterification to 38.50 MJ/kg. It can release a higher amount of heat energy during fuel combustion, which improves engine performance^57,58^. The percentage of unsaturated FFA present in the bio-oil was measured as the iodine number. The Trichosanthes cucumerina bio-oil has an iodine number of 82.4. The degree of unsaturation affects the thermal stability of the fuel and results in carbon deposits^59^. The saponification values were calculated as the amount of potassium hydroxide required for the complete hydrolysis per gram of bio-oil. The saponification value of the Trichosanthes cucumerina bio-oil obtained as 121.37 mg KOH/g. In the other reported works, the saponification values of 192 and 212 mg KOH/g were obtained for Calophyllum inophyllum L. and Ulva lactuca seeds respectively^24,60^ which are higher than the value obtained in this research. The higher saponification values may result in corrosion problem in the diesel engine^61^. The cetane number of 51, 35 and 44 was obtained for diesel, TCO and TCB respectively. Higher cetane number of TCB entails shorter ignition delay leads to improved diffusion part of combustion equated with premixed phase^39,62^.

## Conclusion

Five different solvents were used to synthesis the bio-oil from Trichosanthes cucumerina seeds. The hexane was found to be the better solvent as compared with other solvents. The maximum bio-oil yield was achieved of 28.4 ± 0.4% at a temperature of 68 °C, 0.21 mm crushed seed size, 6% moisture content, 270 min extraction time, and 6:1(ml/g) of solvent to seed ratio. The FTIR analysis shows the higher percentage of oxygen content and less sulphur content in the trichosanthes cucumerina bio-oil. The GC–MS results are seen with the saturated (43.27%) and unsaturated fatty acids (61.14%). The biodiesel was produced from trichosanthes cucumerina bio-oil through the three stages of the trans-esterification process, which produced 93.4 ± 0.2% of biodiesel. The physicochemical properties of the trichosanthes cucumerina bio-oil and biodiesel were analyzed using AOAC and ASTM standards. The biodiesel properties obtained (heating value—38.74 MJ/kg, kinematic viscosity—4.26 cSt and cetane number—44) were closer to diesel fuel, and it can be considered in the diesel engine as an efficient alternative source.

## Data Availability

The datasets generated during and/or analyzed during the current study are available from the corresponding author on reasonable request.

## References

[1] Balamurugan T, Arun A, Sathishkumar GB (2018). Biodiesel derived from corn oil: a fuel substitute for diesel. Renew. Sustain. Energy Rev..

[2] Rajkumar S, Thangaraja J (2019). Effect of biodiesel, biodiesel binary blends, hydrogenated biodiesel and injection parameters on NOx and soot emissions in a turbocharged diesel engine. Fuel.

[3] Dhinesh B, Ambrose YM, Kalaiselvan C, Krishnamoorthy R (2018). A numerical and experimental assessment of a coated diesel engine powered by high-performance nano biofuel. Energy Convers. Manag..

[4] Balaji G, Cheralathan M (2013). Potential of various sources for biodiesel production. Energy Sources Part A Recov. Util. Environ. Eff..

[5] Atabani AE, Silitonga AS, Ong HC, Mahlia TMI, Masjuki HH, Badruddin IA, Fayaz H (2013). Non-edible vegetable oils: A critical evaluation of oil extraction, fatty acid compositions, biodiesel production, characteristics, engine performance and emissions production. Renew. Sustain. Energy Rev..

[6] Voloshin RA, Rodionova MV, Zharmukhamedov SK, Veziroglu TN (2016). Review: Biofuel production from plant and algal biomass. Int J Hydrogen Energy..

[7] Pradhan P, Raheman H, Padhee D (2014). Combustion and performance of a diesel engine with preheated Jatropha curcas oil using waste heat from exhaust gas. Fuel.

[8] Zhang L, Wu H, Yang F, Zhang J (2015). Evaluation of Soxhlet extractor for one-step biodiesel production from Zanthoxylum bungeanum seeds. Fuel Process. Technol..

[9] Luque de Castro MD, Priego-Capote F (2010). Soxhlet extraction: past and present panacea. J. Chromatogr. A..

[10] Dutta R, Sarkar U, Mukherjee A (2014). Extraction of oil from Crotalaria Juncea seeds in a modified Soxhlet apparatus: physical and chemical characterization of a prospective bio-fuel. Fuel.

[11] Vijayakumar C, Ramesh M, Murugesan A, Panneerselvam N (2016). Biodiesel from plant seed oils as an alternate fuel for compression ignition engines: a review. Environ. Sci. Pollut. Res..

[12] Shiu PJ, Gunawan S, Hsieh WH, Kasim NS, Ju YH (2010). Biodiesel production from rice bran by a two-step in-situ process. Bioresour. Technol..

[13] Nanthagopal K, Ashok B, Saravanan B, Mathew S, Chandra S (2018). Effect of next generation higher alcohols and Calophyllum inophyllum methyl ester blends in diesel engine. J Clean Prod..

[14] Khamil KN, Mohd Sabri MF, Yusop AM (2019). Study on novel nano mahua methyl ester powered DI diesel engine preheated with a thermoelectric waste heat recovery system. Energy Sources Part A Recov. Util. Environ. Eff..

[15] Adekunle AS, Adekunle J, Oyekunle O, Obisesan OR, Ojo OS, Ojo OS (2016). Effects of degumming on biodiesel properties of some non-conventional seed oils. Energy Rep..

[16] Sayyar S, Abidin ZZ, Yunus R, Muhammad A (2009). Extraction of oil from jatropha seeds-optimization and kinetics. Am. J. Appl. Sci..

[17] Subramanian N, Mahendradas DK, Kasirajan R, Sahadevan R (2015). Bio-oil separation from potential non-edible urban waste source Putranjiva roxburghii. Sep. Sci. Tech..

[18] Ali MA, Al-hattab TA, Al-hydary IA (2015). Extraction of date palm seed oil (phoenix dactylifera) by soxhlet apparatus. Int. J. Adv. Eng. Technol..

[19] Silva C, Garcia VAS, Zanette CM (2016). Chia (*Salvia hispanica* L.) oil extraction using different organic solvents: oil yield, fatty acids profile and technological analysis of defatted meal. Int. Food Res. J..

[20] Yuvarani M, Kubendran D, Salma Aathika AR, Karthik P, Premkumar MP, Karthikeyan V, Sivanesan S (2017). Extraction and characterization of oil from macroalgae Cladophora glomerata. Energy Sources Part A Recovery Util Environ. Eff..

[21] Jisieike CF, Betiku E (2020). Rubber seed oil extraction: effects of solvent polarity, extraction time and solid-solvent ratio on its yield and quality. Biocatal. Agric. Biotechnol..

[22] Degfie TA, Mamo TT, Mekonnen YS (2019). Optimized biodiesel production from waste cooking oil (WCO) using calcium oxide (CaO) nanocatalyst. Sci. Rep..

[23] Nanthagopal K, Ashok B, Saravanan B, Mathew S, Chandra S (2018). Effect of next generation higher alcohols and Calophyllum inophyllum methyl ester blends in diesel engine. J. Clean. Prod..

[24] Suganya T, Renganathan S (2012). Optimization and kinetic studies on algal oil extraction from marine macroalgae Ulva lactuca. Bioresour. Technol..

[25] Kasirajan R, Pandian S, Tamilarasan S, Sahadevan R (2014). Lipid extraction from natural plant source of Adenanthera pavonina using mixed solvent by superheated extractor. Korean J. Chem. Eng..

[26] John Abraham AP, Chindhanaiselvam A, Subramanian N, Mahendradas D (2017). Biodiesel from Indian laburnum (cassia fistula) kernal oil.. Energy Sources Part A Recov. Util. Environ. Eff..

[27] Murugesan K, Sahadevan R (2017). Optimization of nonedible oil extraction from Cassia javanica seeds.. Energy Sources Part A Recov. Util. Environ. Eff..

[28] Omari A, Mgani QA, Mubofu EB (2015). Fatty acid profile and physico-chemical parameters of castor oils in Tanzania. Curr. Opin. Green Sustain. Chem..

[29] Kalavathy G, Baskar G (2019). Synergism of clay with zinc oxide as nanocatalyst for production of biodiesel from marine Ulva lactuca. Bioresour. Technol..

[30] Theresa V, Ernest Ravindran RS, Ajith Kumar R, Pandian K, Renganathan S (2017). Novel approach to produce oil from non-edible seeds of Indigofera colutea.. Energy Sources Part A Recov. Util. Environ. Eff.

[31] Farsie A, Singh MS (1985). Energy Models for Sunflower Oil Expression. Trans ASAE..

[32] Fadhlullah M, Widiyanto SN, Restiawaty E (2015). The potential of nyamplung (*Calophyllum inophyllum* L.) seed oil as biodiesel feedstock: effect of seed moisture content and particle size on oil yield. Energy Procedia..

[33] Orhevba BA, Chukwu O, Osunde ZD, Ogwuagwu V (2013). Influence of moisture content on the yield of mechanically expressed neem seed kernel oil. Acad. Res. Int..

[34] Al-Sumri A, Al-Siyabi N, Al-Saadi R, Al-Rasbi S, Al-Dallal A (2016). Study on the extraction of date palm seed oil using soxhlet apparatus. Int. J. Eng. Res..

[35] Qian J, Wang F, Liu S, Yun Z (2008). In situ alkaline transesterification of cottonseed oil for production of biodiesel and nontoxic cottonseed meal. Bioresour. Technol..

[36] Shuit SH, Teong K, Harun A, Yusup S (2010). Reactive extraction and in situ esterification of Jatropha curcas L. seeds for the production of biodiesel. Fuel..

[37] Govindhan P, Karthikeyan M, Kumar MD (2019). Environmental Effects Extraction of bio-oil from non-edible novel source Senna occidentalis seeds. Energy Sources Part A Recov. Util. Environ. Eff..

[38] Theresa V, Ernest Ravindran RS, Ajith Kumar R, Pandian K, Renganathan S (2017). Novel approach to produce oil from non-edible seeds of Indigofera colutea.. Energy Sources Part A Recov. Util. Environ. Eff..

[39] Keera ST, El Sabagh SM, Taman AR (2018). Castor oil biodiesel production and optimization. Egypt. J. Pet..

[40] Mohamad M, Ali MW, Ripin A, Ahmad A (2013). Effect of extraction process parameters on the yield of bioactive compounds from the roots of *Eurycoma Longifolia*. Jurnal Teknologi (Sci & Eng.).

[41] Denery JR, Dragull K, Tang CS, Li QX (2004). Pressurized fluid extraction of carotenoids from Haematococcus pluvialis and Dunaliella salina and kavalactones from Piper methysticum. Anal. Chim. Acta..

[42] Kostic MD, Jokovic NM, Stamenkovic OS, Rajkovic KM, Milic PS, Veljkovic VB (2013). Optimization of hempseed oil extraction by n-hexane. Ind. Crops. Prod..

[43] Shah Z, Cataluna Veses R, Silva RD (2016). GC-MS and FTIR analysis of bio-oil obtained from freshwater algae (spirogyra) collected from Freshwater. Int. J. Environ Agric Res..

[44] Sutrisno B, Hidayat A (2018). Pyrolysis of palm empty fruit bunch: Yields and analysis of bio-oil. MATEC Web Conf..

[45] Voli V, Singh RK (2012). Production of bio-oil from de-oiled cakes by thermal pyrolysis. Fuel..

[46] Sharma V, Garg G, Alam A (2014). Extraction and characterization of industrially valuable oil from *Eruca sativa* (L.) Mill through FTIR and GC-MS analysis. Am. J. Biol. Chem..

[47] El Farissi H, Lakhmiri R, El Fargani H, Albourine A, Safi M (2017). Valorisation of a forest waste (Cistus Seeds) for the production of bio-oils. J. Mat and Environ Sci..

[48] Mulimani HV, Navindgi MC (2016). Analysis of physiochemical properties of de-oiled neem seed cake for their suitability in producing bio-oil. Int. J. Mech. Sci..

[49] Tayh SA, Muniandy R, Hassim S, Jakarni F (2017). Aging and consistency characterization of bio-binders from domestic wastes. Int. J. Appl. Eng. Res..

[50] Shah Z, Cataluña Veses R, Silva RD (2016). GC-MS and FTIR analysis of bio-oil obtained from freshwater algae (spirogyra) collected from Freshwater. Int. J. Environ. Agric. Res..

[51] Aigbodion AI, Bakare IO (2005). Rubber seed oil quality assessment and authentication. J. Am. Oil Chem. Soc..

[52] Vlachos N, Skopelitis Y, Psaroudaki M, Konstantinidou V, Chatzilazarou A, Tegou E (2006). Applications of Fourier transform-infrared spectroscopy to edible oils. Anal. Chim. Acta.

[53] Refaat AA (2009). Correlation between the chemical structure of biodiesel and its physical properties. Int. J. Environ. Sci. Tech..

[54] Shahabuddin M, Kalam MA, Masjuki HH, Bhuiya MMK, Mofijur M (2012). An experimental investigation into biodiesel stability by means of oxidation and property determination. Energy..

[55] Knothe G, Steidley KR (2005). Kinematic viscosity of biodiesel fuel components and related compounds. Influence of compound structure and comparison to petrodiesel fuel components. Fuel.

[56] Mort RA, Spooner CE (1940). The calorific value of carbon in coal: the Dulong relationship. Fuel.

[57] Ashraful AM, Masjuki HH, Kalam MA, Rizwanul Fattah IM, Imtenan S, Shahir SA, Mobarak HM (2014). Production and comparison of fuel properties, engine performance, and emission characteristics of biodiesel from various non-edible vegetable oils: a review. Energy Convers. Manag..

[58] Yusuf NNAN, Kamarudin SK, Yaakub Z (2011). Overview on the current trends in biodiesel production. Energy Convers. Manag..

[59] Tubino M, Aricetti JA (2013). A green potentiometric method for the determination of the iodine number of biodiesel. Fuel.

[60] Bhuiya MM, Rasul M, Khan M, Ashwath N, Mofijur M (2020). Comparison of oil extraction between screw press and solvent (n-hexane) extraction technique from beauty leaf (*Calophyllum inophyllum* L.) feedstock. Ind. Crops. Prod..

[61] Muhammad A, Ayub M, Zeb A, Wahab S, Khan S (2013). Physicochemical analysis and fatty acid composition of oil extracted from olive fruit. Int. J. Basic Appl. Sci..

[62] Dhinesh B, Lalvani JI, Parthasarathy M, Annamalai K (2016). An assessment on performance, emission and combustion characteristics of single cylinder diesel engine powered by Cymbopogon flexuosus biofuel. Energy Convers. Manag..

